# Prevalence of Breakfast Skippers among Tunisian Preschool and School Children and Association with Weight Status: A Cross-Sectional Study

**DOI:** 10.3390/children10020392

**Published:** 2023-02-16

**Authors:** Darine Dogui, Radhouene Doggui, Jalila El Ati, Myriam El Ati-Hellal

**Affiliations:** 1 Nutrition Surveillance and Epidemiology in Tunisia Research Laboratory, National Institute of Nutrition and Food Technology, Tunis 1007, Tunisia; 2 Centre de Formation Médicale du Nouveau-Brunswick, Université de Sherbrooke, Moncton, NB E1A 7R1, Canada; 3 Department of Family and Emergency Medicine, Université de Sherbrooke, Sherbrooke, QC J1K 2R1, Canada; 4 Laboratory Materials Molecules and Applications, Preparatory Institute for Scientific and Technical Studies, University of Carthage, Tunis 2070, Tunisia

**Keywords:** breakfast, frequency, quality, weight status, Tunisian children

## Abstract

Breakfast is considered the most important meal of the day. This study aimed to assess breakfast frequency and quality in Tunisian children and to determine the relationship between breakfast skipping and the weight status of the children. A total of 1200 preschool and school children aged 3 to 9 years were randomly selected under a cross-sectional design. Breakfast habits and socio-economic characteristics were collected using a questionnaire. Participants who consumed breakfast less than five times the previous week were categorized as breakfast skippers. The other breakfast consumers were considered as non-skippers. The overall prevalence of breakfast skipping in Tunisian children was 8.3% and 83% of them consumed breakfast all the weekdays. At least two out of three children had a poor breakfast quality. Only 1% of children consumed breakfast in accordance with the composition guidelines. No relationships between breakfast skipping and weight status were detected in this study after adjustment for age, sex and all socio-economic factors (OR = 1.16, 95% CI = 0.72–1.89, *p* = 0.541). Further school-based interventions should be implemented to improve breakfast quality and to promote a healthy weight in Tunisian children.

## 1. Introduction

The prevalence of overweight and obesity has become a major public health problem in recent years, with a considerable increase both in developed and developing countries. Global data showed that the prevalence of overweight and obesity almost quadrupled, from 4% to 18%, between 1975 and 2016 among children and adolescents aged 5–19 years. Furthermore, thirty-eight million children under 5 years were overweight or obese in 2019 [1].

The obesity epidemic is a complex problem, linked to a range of factors, including genetic, environmental, social, and cultural factors [2]. Dietary habits are among the main factors involved in the regulation of the overall energy balance and could influence children’s body weight [3,4,5]. The nutrition transition corresponds to the shift from dietary patterns based on culinary preparations, made from fresh or minimally processed foods, to those based on ultra-processed foods, marked by an increased consumption of animal products, saturated fats, sugar, salt and a deterioration in the consumption of fiber-rich products [6,7,8,9]. This transition has been particularly pronounced in developing countries, where rapid economic growth and urbanization have led to a change in food consumption patterns [10].

In Tunisia, overweight affected 16.5% of children under 5 years in 2020, versus 4.6% in 2000 [11]. Tunisia has experienced a rapid nutrition transition during the last decades. One of the dietary habits that has been affected during this nutrition transition and which could influence children’s body weight is breakfast consumption. Indeed, the association between breakfast frequency and body mass index (BMI) revealed controversial results. As several research studies reported a decrease in BMI with a regular breakfast consumption during childhood [12,13,14,15,16], some studies did not find any significant association between breakfast frequency and weight status [17,18]. These conflicting results could be attributed to significant differences in age, ethnicity, physical activity or socio-economic status of the studied populations or also to variability in the adopted methodologies, such as the time of observation of the research studies [5]. For example, Monzani et al. (2019) [19] found a lack of association between skipping breakfast and the risk of being overweight or obese in infants compared to older children. Moreover, Hopkins et al. (2017) [20] found no association between breakfast consumption and BMI percentile in a sample of low-income African American youth.

The negative association between breakfast consumption and obesity has been attributed to different factors. Indeed, breakfast is the first meal eaten after a long period of fasting and it has a high potential to reduce the obesity risk via the activation of metabolic mechanisms, e.g., increase of postprandial energy expenditure, reduction of ghrelin secretion (a hormone that exerts power on appetite) and provision of a sufficient intake of macro- and micronutrients to maintain body weight [21,22,23].

It has also been reported that breakfast has beneficial effects on memory recall, mood, intellectual performance, school attendance, physical exercise and speaking fluency [24,25,26,27]. The quality of breakfast is also important and the consumption of a high-quality breakfast, including the three major food groups such as grains, fruits and milk products, can improve the whole-diet quality of children [25,28].

The aim of this study was to determine the prevalence of breakfast skippers among Tunisian preschool and school children as well as the quality of consumed breakfast. The association of breakfast frequency with body weight status was also assessed.

## 2. Materials and Methods

### 2.1. Study Design and Participants

This cross-sectional study was conducted from April to May 2017 and concerned a sample of 1200 children aged between 3 and 9 years old and living in the Greater Tunis region. This region includes four governorates (Tunis, Manouba, Ariana and Ben Arous) and is mainly an urban area with an urbanization rate of 93%. The subjects of this study were randomly selected from primary schools and kindergartens on the basis of a two-stage stratified clustered sampling design carried out by the National Institute of Statistics. Stratification was made according to the four governorates and the urban/rural areas. At the first stage, 30 primary schools and 30 kindergartens were selected from the initial sampling frame. At the second stage, 20 children were systematically pinched from each educational institution. The only inclusion criteria were to be aged between 3 and 9 years old and to attend to a kindergarten or a primary school, as well as to have a parent consent. The detailed sample size calculation has been published elsewhere [29].

### 2.2. Socio-Economic Characteristics

The socio-demographic data, including the living area and the parents’ educational level, were collected by trained interviewers using a questionnaire. A household economic score was assessed using an asset-based proxy and divided into tertiles expressing “high”, “medium” or low economic level [30].

### 2.3. Anthropometric Measurements

Anthropometric measurements were carried out according to standard procedures [31]. Body height was recorded to the nearest 0.1 cm using a wall-mounted stadiometer (Person-check^®^, Kirchner and Wilhelm, Germany) and weight was measured to the nearest 0.1 kg with a calibrated scale (Detecto, Webb City, MO, USA). Body mass index (BMI) for-age z-scores were computed as the ratio of the body weight to the body height squared expressed as kg/m². Underweight (BMI-for-age Z-score < −2), overweight (BMI for-age Z-score > +1), obesity (BMI-for-age Z-score > +2) and stunting (height-for-age Z-score < −2) were calculated based on the WHO criteria [32].

### 2.4. Breakfast Skippers

The age at which children and adolescents can accurately report their food consumption without parental assistance is unclear in lower middle-income countries [33]. Although some research from high-income countries has indicated that children between 8 and 12 years can reliably report their food intake, cognitive abilities gradually increase over this time period, which may constrain their ability to self-report before age 12 years [34,35]. In Europe, it has been recommended that children up to 14 years should report food intake with parental assistance [36]. That is why, for our survey on children aged 3–9 years old, we used face-to-face parent interviews to report their young child’s intake during home visits or at school after programming a meeting. Trained dietitians recorded data on the diet history of the week preceding the survey [37]. Breakfast consumption was defined as an eating occasion that occurred between 5 AM and 10 AM on the weekdays and 5 AM and 11 AM on weekends [38].

Breakfast skipping has been defined in several ways in the literature [26]. One recurrent definition of breakfast non-skippers or regular breakfast consumers is that of subjects who consumed breakfast five times the previous week or more (5 to 7 times) [12,15,39,40]. It was adopted in our study. The remaining breakfast consumers were categorized in the literature either into two groups (breakfast skippers, having breakfast less than three times a week (0 to 2 times), and breakfast semi-skippers, having breakfast from three to four times a week) [12,15] or into a group of breakfast skippers eating breakfast less than five times a week (0 to 4 times) [39,40]. In our case, we chose the last definition for breakfast skippers as we preferred dividing the whole breakfast consumers into two groups for statistical analysis. Briefly, subjects who consumed breakfast less than five times the previous week were categorized as breakfast skippers and those who had breakfast five times a week or more were considered as non-skippers in our study.

### 2.5. Breakfast Quality

To assess the overall quality of breakfast, a score based on the consumption of dairy products, cereals and fruits was developed [41]. The breakfast is “ideal” and rated 1 if it includes at least one product from the three groups; the breakfast is “satisfactory” and rated 2 if only one food group is missing; the breakfast is “poor” and rated 3 if two food groups are missing; the breakfast is “very poor” and rated 4 if three food groups are missing.

### 2.6. Ethical Approval

All applicable institutional and governmental regulations on the ethical use of human volunteers were respected. The survey protocol was reviewed and approved by the Ethics Committee on Human Research of the National Institute of Nutrition and Food Technology (INNTA) on 13 January 2017 and by the National Council of Statistics in Tunisia (Visa n° 03/2017) on 15 March 2017. Further, verbal consent was obtained from all the parents and assent was given by children aged six years and above, after being thoroughly informed about the survey’s purpose, risks and benefits.

### 2.7. Data Management and Statistical Analysis

Data entry was performed in duplicate using Epidata software version 3.1 [42]. Taking into account the complexity of the sampling design, the Stata 16 svy function was used for data analysis. Interval variables were expressed as weighted means and categorical variables as proportions. A chi-squared test was used to measure whether there is a relationship between breakfast skipping and co-factors. Linear regression was used to examine the association between skipping breakfast (exposure variable) and being overweight or obese (outcome or dependent variable) after adjustment for age, sex and socio-economic characteristics of subjects. The probability level was set at 0.05.

## 3. Results

A total of 1164 children aged 3–9 years participated in the survey. The non-participation rate was 3%. Table 1 shows the anthropometric and socio-economic characteristics of children according to age groups. The mean age of children was 6.26 ± 0.06 years. The number of boys was equal to that of girls. More than a third of children had overweight or obesity, especially those aged 6–9 years. The majority of parents had high school or more. Households were equally distributed between socio-economic levels (Table 1).

The socio-economic and anthropometric characteristics of Tunisian children relative to breakfast skipping are presented in Table 2. Results showed that 8% of preschool children vs. 8.6% of school children skipped breakfast more than two times a week. Significant differences were noticed between the two age groups (*p* < 0.005). There were no significant differences between breakfast skippers and non-skippers relative to sex or parents’ education level. Children who suffered from underweight, overweight, obesity or stunting were more likely to skip breakfast than the other groups of children.

At least two out of three children had a poor breakfast quality (Table 3). Only 1% of children consumed breakfast in accordance with the composition guidelines, including at least one dairy product, cereals and fruits. With respect to age, there was a significant decrease in breakfast quality from preschool to school children (*p* < 0.05). No significant differences in adherence to guidelines were noticed between boys and girls. Overweight children were more likely to consume a mediocre breakfast quality than non-overweight ones (25.2% of overweight children vs. 8% of non-overweight ones, *p* < 0.0001).

The results of a multi-regression analysis showed a significant association between breakfast skipping and being overweight or obese for an unadjusted model (OR = 2.86, 95% CI = 2.00–4.08, *p* < 0.0001). After adjustment for sex and all socioeconomic factors, this association became not significant (OR = 1.16, 95% CI = 0.72–1.89, *p* = 0.541) (Table 4)

## 4. Discussion

In this study, 8.3% of children aged 3–9 years skipped breakfast more than two times a week and about 87% of them consumed breakfast all the weekdays. The prevalence of breakfast skipping in Tunisian preschool and school children is relatively low compared to that of other research studies. This result could be explained by the nutritional habits of the Tunisian population as well as the several national school-based initiatives that encourage the consumption of a healthy diet by Tunisian children for the prevention of obesity and chronic diseases [43,44]. According to Tee et al. (2018), 20.1% of Malaysian children aged 6–12 years skipped breakfast at least two times a week [12]. A similar frequency of breakfast skipping was observed in 61.2% of Saudi children attending public schools and 63.2% of Indonesian school-aged children [45,46]. Abebe et al. (2022) reported that 38.1 % of Ethiopian school-aged children skipped breakfast more than three times a week [47]. In Iran, the overall prevalence of skipping breakfast among school-aged children was 21.6% [48]. In the United States, 20% of children who consumed no food or beverages, excluding water, at breakfast were considered as breakfast skippers [49]. Generally, the prevalence of breakfast skippers reported in the literature is highly variable (from 0.7% to 74.7%) and it depends on the definition of breakfast skipping used [19].

The prevalence of skipping breakfast among Tunisian children increased with age. This is consistent with previously reported research and could be attributed to an increased autonomy in choosing or purchasing foods or to changes in children’s behavior that are affected by peers [5,20,50,51].

The gender of Tunisian participants and their parents’ education level did not influence the prevalence of breakfast skipping. This result is in contrast with the general findings stating that girls and children with lower parental education presented a higher prevalence of breakfast skipping than the remaining groups [47,49,52,53,54]. The gender differences in breakfast habits could be due to girls’ misconception that skipping meals could make them thinner. This tendency is more common in adolescent girls than in female children. In our study, the age of children did not exceed nine years. This characteristic could explain the similar rates of breakfast skippers among boys and girls. As regards parental education, this factor could significantly affect the eating habits of children, especially if parents are not aware of the benefits of regular breakfast consumption for their child’s health.

The quality of breakfast in the majority of Tunisian children was poor and decreased with age. Only 1% of children consumed a high-quality breakfast, including at least one product from the three major food groups (bread/cereal, dairy products, and fruit). Similar results were reported by Gotthelf et al. (2017) about primary school students from Argentina, of whom 0.8% had a high-quality breakfast and 70.1% had a poor-quality breakfast [41]. Hallström et al. (2012) found that 4% of European adolescents had a breakfast with products from all three of the target food groups [55]. Likewise, 4.8% of Polish children consumed a high-quality breakfast [50]. It has been proved that the quality of breakfast can affect children’s mental health and cognitive function [25,27]. In addition, a good quality breakfast is associated with improved diet quality throughout the day [56]. Ferrer-Cascales et al. (2018) found that Spanish adolescents eating a good-quality breakfast had better health-related quality of life (HRQOL) and lower levels of stress and depression than those who consumed a poor- or very-poor-quality breakfast. Moreover, breakfast skippers presented better HRQOL and lower levels of depression than eaters of a very-poor-quality breakfast. These results reveal the importance of having a good-quality breakfast rather than consuming breakfast regularly [25]. Thus, school-based interventions should be imbedded to enhance breakfast quality in children.

As reported in our previously published work from the same survey [9], ultra-processed food contributed widely to the daily fat, saturated fatty and trans fatty acids intake (≥29%). These ultra-processed foods are often low in fiber, vitamins and minerals, and high in calories [57]. A recent study published by Steele et al. (2017) found that a diet high in ultra-processed foods was associated with lower nutrient intake and higher levels of obesity in children [57]. This is because these foods displace other, healthier foods in the diet and can lead to an overconsumption of calories, leading to weight gain. The health effects of a diet high in ultra-processed foods are not limited to just weight gain and obesity. In children, a diet high in ultra-processed foods has also been linked to poor cognitive development and an increased risk of chronic disease later in life [58]. The growing trend of ultra-processed food consumption among children represents a serious threat to global public health [59].

In this study, we found that the prevalence of breakfast skipping among overweight or obese children was significantly higher than that referred to their normal peers. Results of logistic regression analysis showed a positive association between skipping breakfast and prevalence of overweight or obesity in preschool and school children for the unadjusted model. However, after adjustment for sex and all socio-economic factors, this association disappeared. Global data on the relationship between weight status and breakfast frequency are controversial. According to the meta-analysis of 45 observational studies conducted by Ma et al. (2020) [14], skipping breakfast increased the risk of overweight or obesity by 48% in cross-sectional studies and 44% in cohort studies. Likewise, Vik et al. (2016) [60] reported, in a cross-sectional, school-based survey conducted in eight European countries, that having a regular family breakfast was inversely associated with overweight (OR = 0.78 (95% CI 0.67–0.91)). Other literature surveys did not find any association between breakfast consumption and weight status [17,61]. Focusing on interventional studies, the systematic review conducted by Ricotti et al. (2021) [5] reported controversial findings between longitudinal studies and randomized controlled trials concerning the association of breakfast skipping with a high risk of being overweight or obese. The authors attributed these differences to several factors, such as age, ethnicity, physical activity, socio-economic status or time of observation [5]. In Tunisia, the implementation of intervention programs for school children improved good diet behaviors (such as daily breakfast) in the intervention group. However, no significant effect on the body composition of children was observed [43,44]. Therefore, more elaborate, multidisciplinary interventions were recommended to have a tangible effect on the incidence of overweight and obesity [43].

The association between breakfast skipping and obesity has significant implications for public health and highlights the need for effective strategies to promote breakfast consumption and reduce obesity. One potential implication is the need to improve access to healthy breakfast options, especially for individuals who may face barriers to eating breakfast, such as those with limited financial resources or those who live in food deserts. Providing access to healthy and affordable breakfast options can help to reduce the prevalence of breakfast skipping and the associated risk of obesity. Another implication is the importance of education and promotion of the benefits of eating breakfast. This can involve efforts to increase awareness about the role of breakfast in promoting overall health and reducing the risk of obesity and other chronic diseases. Education programs and campaigns could be targeted to different populations, such as children and adolescents, who may be particularly vulnerable to the effects of breakfast skipping. Furthermore, the findings of the study could inform public health policy- and decision-making by highlighting the need for targeted interventions aimed at reducing breakfast skipping and promoting healthy breakfast consumption. For example, school-based programs could be developed to encourage children to eat breakfast and to provide healthy breakfast options in schools [62].

The strengths of our study include the large sample of children aged between 3 and 9 years old living in the Greater Tunis region. This region is the most cosmopolitan in Tunisia and tends to be representative of the general population. Furthermore, adjustment has been realized for several confounding variables, e.g., socio-demographic factors.

The present study has some limitations that should be considered. Our results are based on a cross-sectional study, so that we cannot establish a temporal relationship between exposure (breakfast skipping) and outcome (overweight and obesity), making it difficult to determine cause and effect. It is possible that the outcome is affecting the exposure, rather than the other way around, which can lead to biased results. Moreover, the lack of a standardized definition of breakfast skipper makes it difficult to compare and generalize findings across studies, and highlights the need for clearer definitions and criteria in research on breakfast skipping.

## 5. Conclusions

The prevalence of breakfast skippers among Tunisian preschool and school children was relatively low compared to the global data. However, the quality of breakfast was poor for the majority of children. The association between breakfast skipping and body weight was not detected in this study. Therefore, more intervention studies are needed to encourage the regular consumption of a healthy breakfast and to investigate the relationship between breakfast consumption and adiposity status.

## Figures and Tables

**Table 1:** Characteristics of Tunisian preschool and school children.

	Age Group (Years)		
	3–9	3–5	6–9
Number of children	1164	532	632
Number of boys	582	279	303
Number of girls	582	253	329
Mean age (years)	6.3	4.6	8.1
Mean height (cm)	118.0	107.8	129.1
Mean weight (kg)	23.6	19.1	28.5
BMI (kg/m^2^)	16.6	16.4	16.9
Underweight (%)	3.0	1.2	4.9
Overweight (%)	26.0	21.7	30.6
Obesity (%)	9.9	6.1	14.1
Stunting (%)	1.4	1.6	1.1
Father education level (%)			
No formal education	0.8	0.7	0.9
Primary schooling	22.7	18.1	27.9
Secondary schooling	40.0	40.7	39.2
University level	36.4	40.5	32.0
Mother education level (%)			
No formal education	2.6	1.5	3.9
Primary schooling	20.7	14.2	27.7
Secondary schooling	36.0	35.2	36.9
University level	40.6	49.1	31.5
Socio-economic level (%)			
Low	32.5	28.7	36.6
Medium	34.4	37.6	30.9
High	33.1	33.6	32.5

**Table 2:** Breakfast consumption of Tunisian children according to their socio-economic and anthropometric characteristics.

	Skippers			Non-Skippers			*p*-Value ^1^
	*n*	% ^2^	95% C.I.	*n*	%	95% C.I.	
Age (years)							
3–5	44	8.0	6.0–10.6	488	92.0	89.4–94.0	0.0017
6–9	56	8.6	6.7–11.1	576	91.4	88.9–93.3	
Sex							
Boys	51	8.5	6.5–11.1	531	91.5	88.9–93.5	0.7866
Girls	49	8.1	6.1–10.6	533	91.9	89.4–93.9	
Father education level							
No formal education	00	0.0		10	100.0		0.7028
Primary schooling	25	8.9	6.0–12.9	143	91.1	87.1–94.0	
Secondary schooling	41	8.6	6.3–11.5	422	91.4	88.5–93.7	
University level	31	7.5	5.3–10.5	378	92.5	89.5–94.8	
Mother education level							
No formal education	5	14.7	6.0–31.8	28	85.3	68.2–94.0	0.1807
Primary schooling	28	10.8	7.5–15.2	222	89.2	84.8–92.5	
Secondary schooling	30	7.1	5.0–10.1	391	92.9	89.9–95.0	
University level	35	7.4	5.3–10.2	420	92.6	89.8–94.7	
Underweight							
Yes	7	20.8	10.3–37.7	30	79.2	90.4–93.5	0.0073
No	93	7.9	6.5–9.6	1034	92.1	62.3–89.7	
Overweight							
Yes	57	18.2	14.3–23.0	254	81.8	77.0–85.7	<0.0001
No	43	4.8	3.6–6.5	810	95.2	93.5–96.4	
Obesity							
Yes	26	21.4	14.9–29.8	96	78.6	70.2–85.1	<0.0001
No	74	6.9	5.5–8.6	968	93.1	91.4–94.5	
Stunting							
Yes	4	24.0	9.1–49.7	12	76.0	50.3–90.9	0.0214
No	96	8.1	6.6–9.8	1052	91.9	90.2–93.4	

^1^*p* value for the chi-squared test for breakfast skipping × cofactor. ^2^ Valid percentages per line.

**Table 3:** Breakfast quality of Tunisian children according to age, gender and weight status.

Breakfast Quality	Age (Years)			Gender			Weight Status		
	3–5 %	6–9 %	*p*-Value	Boys %	Girls %	*p*-Value	Non-Overweight %	Overweight %	*p*-Value
Ideal	1.5	0.6	0.034	1.1	1.0	0.94	1.1	0.9	<0.0001
Satisfactory	19.1	14.3		17.4	16.2		17.2	15.6	
Poor	68.6	70.7		68.9	70.4		73.6	58.3	
Very poor	10.8	14.3		12.6	12.4		8.0	25.2	

**Table 4:** Logistic regression analysis of the association between skipping breakfast and being overweight or obese.

Breakfast Consumption	N	Unadjusted Model			Adjusted Model		
		OR ^a^	95% CI ^b^	*p*-Value	OR	95% CI	*p*-Value
Total sample	1164						
Non-Skippers	100	1.00			1.00		
Skippers	1064	2.86	2.00–4.08	<0.0001	1.16	0.72–1.89	0.541
Preschool children	532						
Non-skippers	488	1.00			1.00		
Skippers	44	3.27	1.83–5.86	0.0001	0.73	0.26–2.05	0.546
School children	632						
Non-skippers	56	1.00			1.00		
Skippers	576	2.42	1.55–3.80	0.0001	1.57	0.86–2.83	0.139

^a^ Odds ratio. ^b^ 95% sampling design-based confidence interval.
